# Health Literacy and Numeracy in Self-monitoring of Capillary Glycemia: A Systematic Review of Mixed Methods

**DOI:** 10.2174/0115733998344984241203070203

**Published:** 2025-01-09

**Authors:** Luis Angel Cendejas Medina, Maria Girlane Sousa Albuquerque Brandão, Glaubervania Alves Lima, Maria Lucia Zanetti, Ana Emília Pace, Maria del Pilar Serrano Gallardo, Juana Mercedes Gutierrez Valverde, Brena Shellem Bessa de Oliveira, Francisca Elisangela Teixeira Lima, Carla Regina de Souza Teixeira

**Affiliations:** 1 College of Nursing of the University of São Paulo at Ribeirão Preto Campus, Ribeirão Preto, São Paulo, Brasil;; 2 Department of Nursing, Federal University of Ceará, Fortaleza, Ceará, Brasil;; 3 Medical School, Autonomous University of Madrid, Madrid, España, Spain;; 4 Faculty of Nursing, Autonomos University of Nuevo León, Monterrey, Nuevo León, México

**Keywords:** Health literacy, numeracy, diabetes mellitus, blood glucose self-monitoring, mortality, musculoskeletal change

## Abstract

**Objective:**

The aim of this study was to synthesize scientific evidence on the influence of health literacy and numerical knowledge on self-monitoring of capillary blood glucose.

**Methods:**

Adhering to the PRISMA guidelines and the principles of the Joanna Briggs Institute, a comprehensive search was conducted across multiple databases, including CINAHL, Cochrane, Embase, LILACS, PubMed, Scopus, Web of Science, Google Scholar, OPENGREY, and NDLTD. The review included studies published in any language that examined the relationship between HL, numeracy, and SMBG.

**Results and Discussion:**

A total of 12 studies met the inclusion criteria. These studies utilized various assessment tools, such as the Brief Test of Functional Health Literacy in Adults (B-TOFHLA) and the Diabetes Numeracy Test (DNT-15), to evaluate health literacy and numeracy levels. The findings revealed a significant association between adequate HL and numeracy and improved SMBG practices. Specifically, individuals with sufficient health literacy were more likely to monitor their blood glucose levels regularly and make appropriate treatment adjustments based on their readings.

**Conclusion:**

The results indicated that numeracy skills and health literacy are critical determinants of effective SMBG, influencing the frequency and accuracy of self-care practices in diabetes management. These findings highlight the urgent need for educational interventions tailored to enhance these skills, which could lead to improved health outcomes for individuals with diabetes.

## 1 INTRODUCTION

Diabetes Mellitus (DM) is one of the health challenges in the 21^st^ century. According to the International Diabetes Federation (IDF), 537 million people worldwide live with this disease, which is expected to reach 784 million by 2045.-Additionally, DM is among the top ten causes of death globally, responsible for 6.7 million deaths in 2021 [1]. Due to the increase in morbidity and mortality, type 2 diabetes (T2DM) is one of the leading causes of disease burden in many countries. This is primarily attributed to the elevated risk of developing a range of serious health issues characterized by vascular and musculoskeletal changes resulting from persistently high blood glucose levels [2]. Thus, glycemic monitoring is emphasized as a fundamental strategy in preventing diabetes complications, resulting in improved quality of life for people living with this chronic disease [3]. Currently, with the advent of new technologies, Continuous Glucose Monitoring (CGM) through subcutaneously applied sensors, which allow uninterrupted measurement of current and real glucose levels, emerges with new metrics for assessing glycemic control, such as Time in Range (TIR) [4]. However, despite the potential health benefits associated with Self-Monitoring of Blood Glucose (SMBG) and CGM in diabetes treatment, there is high variability in the performance and daily frequency of self-monitoring among individuals, or simply it is not regularly performed. Therefore, adherence to glucose monitoring is generally below ideal [5]. There are still barriers to blood glucose monitoring, such as pain, lack of understanding about blood glucose levels, low self-efficacy in monitoring, low self-esteem, limited social support, and tendencies towards anxiety and depression to complicate treatment. Additionally, effective communication with medical teams is also lacking, often due to insufficient health education and the absence of health insurance plans [4, 6]. Therefore, the success of these new technologies may, however, critically depend on the level of people being educated, capable, and motivated to use them. In this way, one of the main barriers is to be able to interpret each one of these glycemic values, patterns, and trends by people. Therefore, Health Literacy (HL) and Numeracy are necessary to achieve favorable outcomes with this new technology [7]. HL is defined as a set of skills that allows individuals to obtain, process, and use health information to make decisions that favor overall health status [8]. On the other hand, health numeracy is considered part of HL. It has been defined as “the ability to perform basic reading and numerical tasks necessary to function in the healthcare environment” [9, 10]. HL and numeracy in diabetes management are closely intertwined, with numeracy skills critical for effective self-management. Numeracy in diabetes involves tasks such as carbohydrate counting, reading food labels, and accurately administering insulin and medications [11]. Inadequate HL and numeracy can hinder success in self-care, including achieving TIR and other management goals in diabetes [12]. Therefore, assessing HL in individuals is essential before implementing educational interventions aimed at improving self-care outcomes. That is why it becomes critical to track in the literature what has been evidenced so far about the relationship between HL and Numeracy in people living with DM who perform continuous glucose monitoring or capillary glycemic self-monitoring. Thus, the objective of the present study was to synthesize scientific evidence on the influence of health literacy and numerical knowledge on self-monitoring of capillary blood glucose.

## 2 MATERIALS AND METHODS

This study is a systematic review of mixed methods (SRMM). This type of review involves gathering findings of effectiveness (quantitative evidence) and the experiences of patients, families, staff, or others (qualitative evidence) to enhance their usefulness for decision-makers [13]. There is a growing focus on the different types of information that guideline developers need when making decisions, such as feasibility, priority, cost-effectiveness, impact on equity, acceptability [14], and patient values and preferences [15]. Integrating data in response to these different types of questions into a single synthesis is useful for guideline development groups and decision-makers.

Although there is a degree of complexity in conducting SRMM, the central intention is to combine quantitative and qualitative data (from primary studies) or to integrate quantitative evidence and qualitative evidence to create a breadth and depth of understanding that can confirm or challenge evidence and ultimately answer the review question(s) posed. Therefore, the numerical data inherent to the positivist paradigm can support or endorse the equally important opinions and perspectives presented in interpretative and critical paradigms and vice versa. This has the potential to produce more informative conclusions than those derived from evidence presented in autonomous modes of synthesis, namely systematic reviews of effectiveness and experiential systematic reviews.

This is an SRMM literature study conducted following the Joanna Briggs Institute's (JBI) [16] principles, which was developed according to the Preferred Reporting Items for Systematic Reviews and Meta-Analysis (PRISMA) guidelines [17]. The protocol for this systematic review was submitted and registered in the International Prospective Register of Systematic Reviews (PROSPERO) with the number (CRD42023408045). It is available at: https://doi.org/10.17665/1676-4285.20246719. The present review followed nine chronological steps according to JBI [18].

### 2.1 Formulation of the Guiding Question of the Review

The research guiding question was developed using the PICo strategy, which represents an acronym for Population of interest (People with diabetes mellitus), phenomenon of interest (Functional Health Literacy and Numeracy), and context (Capillary Glycemic Self-Monitoring or Continuous Glucose Monitoring). Thus, the question of the systematic review was: “What is the influence of HL and numeracy in people with DM who perform SMBG or CGM?”.

### 2.2 Inclusion and Exclusion Criteria

Primary studies (theses included) with quantitative, qualitative, or mixed approaches were included, which analyzed the influence of HL and Numeracy in people with DM who performed SMBG or CGM in any publication period and language. Case reports, case series, secondary studies (other reviews), editorials, letters to the editor, books, book chapters, guidelines, expert opinion articles, experience reports, conference proceedings, and abstracts were excluded from this review.

### 2.3 Data Source

The screening of studies took place using the following electronic databases: Cumulative Index to Nursing and Allied Health (CINAHL), Cochrane, Embase, *Literatura Latino-Americana e do Caribe em Ciências da Saúde* (LILACS), PubMed, Scopus, and Web of Science. Additionally, grey literature was explored through Google Scholar, OPENGREY, and The Networked Digital Library of Theses and Dissertations (NDLTD). To construct the search strategy, appropriate controlled vocabulary was used to identify as many documents related to the subject as possible. Thus, controlled descriptors from DeCS/MeSH and their synonyms were used: “diabetes mellitus,” “health literacy,” “numeracy”, “blood glucose self-monitoring,” and “continuous glucose monitoring,” associated with boolean operators ‘AND’ or ‘OR’, grouped and adapted according to the specificities of each database consulted in this review. The full versions of the search strategies can be found at: https://doi.org/10.17665/1676-4285.20246719. Electronic access to all databases occurred on January 16, 2023. After identifying references, duplicate removal was managed using EndNote Basic online reference manager. Subsequently, studies were imported into the Rayyan platform for initial assessment. Two reviewers independently and blindly screened titles and abstracts based on eligibility criteria. Eligible studies underwent full-text review. Any discrepancies between reviewers were resolved by consulting a third reviewer.

### 2.4 Extraction and Analysis Process of Selected Study Information

After selection in Rayyan, the full articles were independently reviewed by two investigators using a predefined form. This form included fields such as authors and year, country/language, objectives, sample characteristics, study settings, instruments used, main results, and level of evidence [19]. Each study underwent rigorous methodological evaluation, and findings were synthesized descriptively. Data extraction focused on addressing the research question and eligibility criteria. Group 1 prioritized information related to interventions, population characteristics, methods, and results. Group 2 emphasized descriptions and specific details about the population, context, culture, and relevant aspects of the phenomenon under review [16].

### 2.5 Data Analysis and Synthesis

The JBI recommends using the segregated convergent approach when quantitative and qualitative evidence can be clearly distinguished [18]. Although the authors aimed to create two distinct syntheses and then integrate them, the findings were primarily quantitative, making qualitative synthesis impossible. Consequently, a single synthesis was created, with data broadly interpreted. Article characteristics and results were presented in tables, while the overall review results, discussion, and final synthesis were presented in text.

### 2.6 Evaluation of Methodological Quality

The risk of bias assessment of the primary studies in the sample utilized JBI tools, which are designed to critically evaluate methodological quality and potential biases in study design, conduct, and analysis. Eligible studies were independently assessed by two reviewers using JBI's critical appraisal tools, with any discrepancies resolved by a third reviewer. Contacting study authors to clarify or obtain missing data was also part of this rigorous evaluation process [16, 20].

## 3 RESULTS

After electronic database searches, 459 records were identified, with 95 duplicates removed. Subsequently, 364 documents underwent title and abstract screening, resulting in 23 studies selected for full-text review. Following full-text assessment, 11 articles were excluded based on eligibility criteria. Ultimately, 12 studies were included in the systematic review sample and underwent qualitative analysis, as illustrated in Fig. (**1**).

Critical evaluation of the methodological quality of the selected articles, shown in Fig. (**2**), revealed the following: 10 articles used the Cross-Sectional Studies Checklist. All 10 (100%) adequately described inclusion criteria (Q1) and provided clear information about patient selection environments (Q2). Eight articles (80%) measured exposure validly and reliably (Q3), and 87.5% used objective, standardized criteria to measure the condition (Q4).

However, two studies did not measure outcomes validly and reliably (Q7), and two did not use appropriate statistical analysis (Q8). One study applied the Qualitative Research Checklist, with only two of the 10 assessment items considered uncertain: the researcher's cultural and theoretical location (Q6) and the researcher's influence on the research (Q7). The study “Randomized Trial of a Literacy-Sensitive, Culturally Tailored Diabetes Self-Management Intervention for Low-Income Latinos” applied the Randomized Controlled Trials Checklist, where 10 out of 12 items evaluated were positively congruent.

The studies, published between 2010 and 2021, predominantly in English (n=11), originated from Indonesia (n=1), USA (n=6), Hungary (n=1), Iran (n=1), Brazil (n=1), Denmark (n=1), and South Korea (n=1). They aimed to identify factors influencing SMBG self-monitoring and explore the impact of health literacy (HL) and numeracy on SMBG (Table **1**).

The sample size of the analyzed studies varied from nine to 4,397 participants. The research settings included public health centers, Centers for Disease Control and Prevention, diabetes associations, diabetes reference centers and/or clinics, university health units, and hospital clinics. The studies used the following data collection sources: Brief Test of Functional Health Literacy in Adults (B-TOFHLA), Diabetes Numeracy Test (DNT-15), Health Literacy for Iranian Adults (HELIA), Diabetes Self-Care Activities Questionnaire (QAD), Health Literacy Questionnaire (HLQ), Summary of Diabetes Self-Care Activities Scale, Newest Vital Sign (NVS), Health Literacy Scale (HLS), Diabetes Empowerment Scale-28 (DES-28), Summary of Diabetes Self-Care Activities (SDSCA), and custom questionnaires. It was identified that HL and numeracy favored the practice of self-monitoring of capillary glycemic at the appropriate frequency and allowed for adjustments to treatment as necessary based on glucose measures.

### 3.1 Main Findings on the Influence of Functional Health Literacy and Numeracy on Blood Glucose Self-monitoring

Three themes were developed on the influence of HL and Numeracy on SMBG in people with DM, presented as follows: “SMBG.” “Evaluation of blood glucose at the recommended frequency,” and “Correct insulin calculation and interpretation based on current blood glucose levels.”

### 3.2 Blood Glucose Self-monitoring

Of the 12 studies included in the review sample, 11 reported impacts on SMBG, with most of these studies conducted with people with T2DM over 15 years. Only one of these studies focused on people using insulin, which shows that the population with T2DM using this medication and performing SMBG needs to be further explored. The scenario where most of these studies were conducted was in health centers, with only one study conducted in a hospital setting.

Most studies evaluated only HL using a validated instrument [21, 22, 24, 26, 28, 31, 32]. One study used its questionnaire [22], and another carried out an intervention through a program aimed at developing HL [29]. However, numeracy was evaluated in only two of these studies [23, 30], which also highlights the lack of measurement of this skill in people with T2DM. Additionally, a large part of the studies showed a positive correlation between HL and SMBG (*p* >0.05) [21, 23, 24, 28, 30, 31].

In the remaining studies, it was observed that people with DM and adequate HL performed SMBG more frequently than those with inadequate HL [24, 29]. This demonstrates the inverse effect where people with inadequate HL did not SMBG. Thus, HL appears to be a limiting factor for not performing this management practice, or it is not perceived as having intrinsic value for its implementation [22, 26, 29].

### 3.3 Evaluation of Blood Glucose at the Recommended Frequency

The results of this mixed-method systematic review also demonstrated that HL not only influences SMBG but also the evaluation at the frequency recommended by healthcare professionals, where this finding was observed in six studies [21, 23, 25, 27, 30] of the 12 that comprise the sample. Thus, people with DM and adequate HL evaluated blood glucose at least once a day, as requested by healthcare professionals, or the number of times requested. It is noteworthy that only in article 12 it was mentioned that the participants, in addition to measuring blood glucose as requested by the professional, recorded the values to keep track and be able to review the different fluctuations of their glucose, which in turn allows for medication adjustments and other therapeutic measures such as carbohydrate intake and physical exercise.

### 3.4 Correct Insulin Calculation and Interpretation Based on Current Blood Glucose Levels

Two studies [23, 30] mentioned understanding and interpreting the appropriate insulin dosage based on blood glucose levels through prior SMBG. It is worth noting (as mentioned in the first theme) that both studies assessed participants' numeracy using the Diabetes Numeracy Test (DNT-15) instrument. Additionally, in both studies, there was a positive correlation between numeracy and SMBG.In this regard, having numerical skills aids in figuring out the necessary insulin dosages and comprehending the readings from the glucometer. Therefore, the presence of HL and numeracy in individuals with T1DM and T2DM influences the use of parenteral medicine and, consequently, the adjustment of SMBG based on perceived blood glucose levels.

The reviewed and included studies indicate a positive correlation between HL and the frequency with which patients perform SMBG. This demonstrates how patients' HL levels directly influence this practice in understanding the importance of SMBG. Furthermore, patients with higher HL followed healthcare professionals' recommendations regarding the frequency and execution of SMBG more accurately, while those with lower HL did not do so as consistently. On the other hand, numeracy is crucial for the proper management of medication based on blood glucose levels and, therefore, directly influences clinical outcomes.

These findings not only fulfill the objective of the review but also offer valuable insights for healthcare professionals seeking to improve adherence and accuracy in SMBG through educational interventions. The evidence confirms that both HL and numeracy are key determinants in the proper management of DM. From this review, targeted interventions can be developed to improve these skills, which could directly impact adherence and the effectiveness of self-monitoring.

## 4 DISCUSSION

The proposed review addresses a gap in our understanding of the influence of HL and numeracy on SMBG and CMG, as health tools and technologies have the potential to transform DM self-management, including among more vulnerable groups.

Understanding the influence of HL and numeracy on these self-management activities is a crucial step. It is emphasized that this is particularly an important time, where access to technology for SMBG and CGM is increasing worldwide [33], especially after the emergence of TIR and Point in Range (PIR) measures, which are obtained through blood glucose values measured in SMBG and CGM, showing as new markers to assess and achieve glycemic control [34].

The results of this systematic review highlight the positive influence of numeracy and HL on SMBG. It was identified that both analyzed factors are critical elements that affect people's ability to understand, interpret, and apply information related to DM management. It has been evidenced that health literacy (HL) and numeracy influence self-care activities and habits, such as daily blood glucose testing [24, 23].

These findings may be related to the fact that people with higher HL have a deeper understanding of information related to their condition and knowledge about the risks associated with hyperglycemia and hypoglycemia. Therefore, they tend to be motivated and capable of adopting self-care behaviors, such as regular Blood Glucose Monitoring (BGM).

Likewise, the literature suggests that knowledge about diabetes and health literacy (HL) affects glucose monitoring and absenteeism from work/school in individuals who start using CGM. As such, it emphasizes the importance of education [7]. Similarly, individuals with adequate HL reported higher levels of understanding and comfort with CGM, suggesting that CGMs should not be withheld from people with low HL. Incorporating baseline HL assessment and offering sensitive training will help optimize the benefits derived from this technology. CGMs are powerful tools for improving clinical outcomes in patients with diabetes, and their benefits should not be underestimated. However, they are technologically advanced, and not all patients may be able to fully benefit from these devices on their own without HL-sensitive materials and in-depth or ongoing training. While patients with low HL may not feel as comfortable or confident using their CGM initially, they should not be excluded from its potential benefits [35].

This makes HL one of the determining factors for SMBG in people with DM [21]. Thus, educational intervention programs and support with dissemination of information related to DM and SMBG can play a crucial role in improving health outcomes and encouraging adherence to regular self-monitoring practice.

Numeracy's influence may be associated with people's ability to understand and interpret the numbers associated with glycemic control. Numerical skills help recognize alert situations and make more assertive decisions based on the numbers presented on the glucometer. In contrast, those with numeracy difficulties may face challenges in understanding the severity of glycemic fluctuations and are less likely to adhere to an effective self-monitoring plan.

Another important aspect was the identification of the effects of HL and numeracy on the evaluation of blood glucose at the recommended frequency [23]. This is a relevant finding, as periodic monitoring of capillary blood glucose is fundamental in the effective control of DM. However, it is important to note that not all people diagnosed with DM regularly adopt this practice.

In the same sense, the literature reports a relationship between numeracy and better HbA1c and other behavioral indicators, such as greater perceived self-efficacy [36-38]. These findings suggest that the ability to interpret and use numbers is directly associated with better outcomes in glycemic control. This makes sense, as people with more developed numerical skills are able to correctly interpret the values presented by the glucometer and adjust their behavior (diet, medications, or exercise) to keep glucose levels under control. At the same time, when a person feels more confident interpreting the numbers, they feel more secure in making decisions about managing the disease independently, which is essential for maintaining effective and continuous monitoring. Both ideas highlight how numeracy not only affects the understanding of glucometer readings but also translates into more effective health behaviors.

Conversely, the literature also shows that HL does not influence this care behavior, such as self-monitoring of blood glucose levels [32, 39]. This may happen because SMBG is only recommended for patients with type 2 diabetes if their diabetes is poorly controlled or if they are taking insulin [38, 40]. As a result, it becomes a management and care activity incorporated in an exacerbated state of the disease. Therefore, it is necessary to address this management activity in the early stages of the disease so that people with type 2 diabetes are aware of the use and utility of SMBG.

Brazilian researchers found that specific dimensions of diet, physical activity, and BGM demonstrate undesirable self-care behavior among Brazilians and explain that people with DM who perform capillary SMBG lack specialized professional follow-up in association with the execution of continuous educational practices to stimulate their literacy and numeracy skills, to increase their effective participation in self-care activities [41].

It is worth noting that in addition to considering the assessment of HL and numeracy, it is necessary to assess socioeconomic conditions and the possibility of acquiring glucose meters in sufficient quantities for periodic glucose monitoring to ensure that all people with DM can effectively manage their condition and improve their quality of life.

It is noteworthy that glucose monitoring consists not only of checking and mapping blood glucose values but also serves as an indicator of the need for insulin dose adjustments. This is an important item, given that in people with DM, the worst dimensions for carrying out the necessary care are medication use and taking insulin injections correctly based on capillary blood glucose values [42].

In the present study, it was identified that numeracy also influences the correct calculation of insulin doses and their interpretation based on blood glucose levels. Higher numeracy has a significant effect on the appropriate insulin dosage, making medication adjustments based on current blood glucose levels [23, 30]. This point highlights the substantial difference in patients' ability to make medication adjustments based on glucose readings, depending on the type of care they receive and greater numerical ability [43].

This also suggests that follow-up in specialized centers, where patients receive specific information about their condition, plays an essential role in individuals' ability to manage their health condition. Therefore, offering health education programs and access to specialized centers can improve patients' ability to deal with medication adjustments based on their health indicators, such as glucose readings in the case of DM, and reduce complications, such as hypo or hyperglycemia, which in the long run can lead to more serious complications.

Given the above, our systematic review has the potential to contribute new knowledge to this audience and health professionals who care for people using SMBG and who intend to measure TIR and implement care strategies based on this metric [34] in addition to helping develop strategies and educational materials that will facilitate the application of these concepts in these self-management activities of DM and other health conditions.

The present systematic review of mixed methods has some limitations that should be considered when interpreting the results. Among these are significant variations in participant profiles across studies, attributable to differences in countries, cultural contexts, educational levels, and access to healthcare, as well as differences in the measurement and evaluation of health literacy, given that different instruments were applied.

Moreover, the heterogeneity of the included studies represents a significant challenge, but this is inherent to this type of systematic review, given the aim of including different methodological designs. The analyzed studies employ varying methodologies, approaches, and measures, which can hinder the comparability of findings and limit the generalization of conclusions. The predominance of cross-sectional studies also restricts the ability to identify temporal sequences or infer causal relationships, as these studies collect data at a single point in time, preventing the analysis of variations over time. Furthermore, the lack of control over confounding factors, such as treatment adherence and prior education in diabetes management, may influence the observed results, limiting the ability to establish conclusive associations between health literacy and self-monitoring practices. It is also important to note the significant variations in participant profiles across studies, attributable to differences in countries, cultural contexts, educational levels, and access to healthcare, as well as differences in the measurement and evaluation of health literacy, given the application of different instruments. Nonetheless, the results presented here remain important for clinical practice.

New qualitative or mixed-methods studies are needed to gain deeper insights into the experiences of people with DM who perform SMBG and CGM under the light of HL and numeracy to ensure the influence of these concepts on this self-care activity, to perceive the weight of these in DM management activities and encourage glycemic control maintenance.

## CONCLUSION

It is concluded that there is a significant influence between HL and numeracy in SMBG in the evaluation of blood glucose at the frequency recommended by professionals and in the correct calculation of insulin doses and their interpretation based on current blood glucose levels obtained from SMBG. Considering the main findings of the present study, healthcare professionals and managers will be able to direct their efforts better to offer personalized education to people with DM, as well as access to enhanced resources and the development of educational strategies that improve users' understanding, literacy, and numeracy, to contribute to glycemic control, quality of life, and reduction of short- and long-term complications.

## CLINICAL IMPLICATIONS OF THE FINDINGS

The main clinical implications of this systematic review on the influence of HL and numeracy in SMBG highlight that individuals with adequate HL tend to perform SMBG more frequently. At the same time, those with inadequate HL show lower adherence. This suggests the need for healthcare professionals to assess patients' HL and adapt educational materials and programs to enhance understanding of SMBG. Patients with higher HL monitor their blood glucose more regularly, facilitating therapeutic adjustments, such as insulin administration and adjustments in diet and physical activity. Digital or analog tools can support those who struggle to follow recommendations, particularly patients with lower HL.

Numeracy also plays a crucial role in adjusting insulin doses. Patients with adequate numerical skills are better able to adjust their doses according to blood glucose levels, while those with low numeracy face difficulties, compromising diabetes control. Therefore, it is essential for healthcare professionals to identify these limitations and provide solutions, such as digital calculators or automatic devices. Moreover, it is recommended that professionals be trained to address different levels of HL and numeracy, develop customized educational tools, and integrate these practices into public health policies to improve control of diabetes in vulnerable populations.

## AUTHORS’ CONTRIBUTIONS

L.A.C.M, M.G.S.A.B., G.A.L., M.L.Z., A.E.P., M.D.P.S.G., J.M.G.V., B.S.B.D.O., F.E.T.L., and C.R.D.S.T. contributed to the research design and implementation, as well as the data analysis and manuscript writing.

## Figures and Tables

**Fig. (1):**
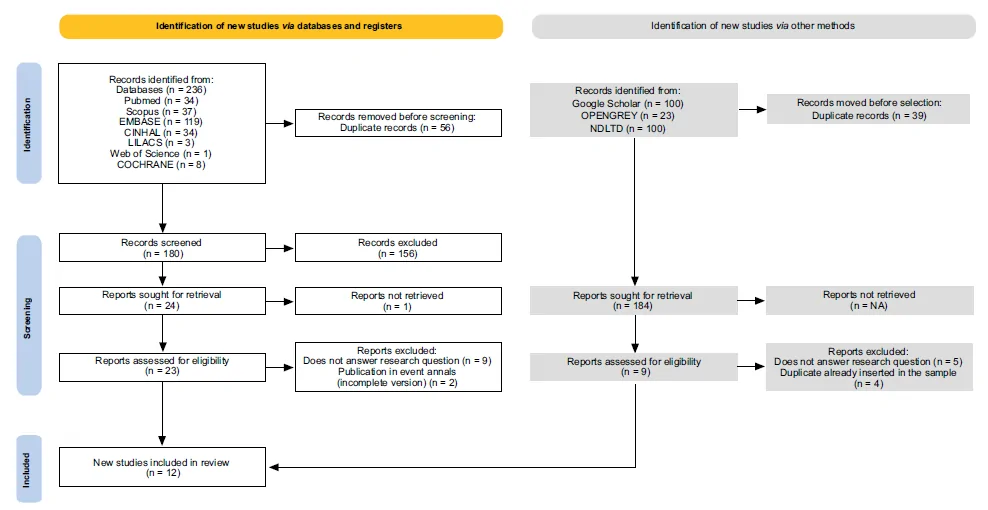
PRISMA flowchart of literature search and selection process.

**Fig. (2):**
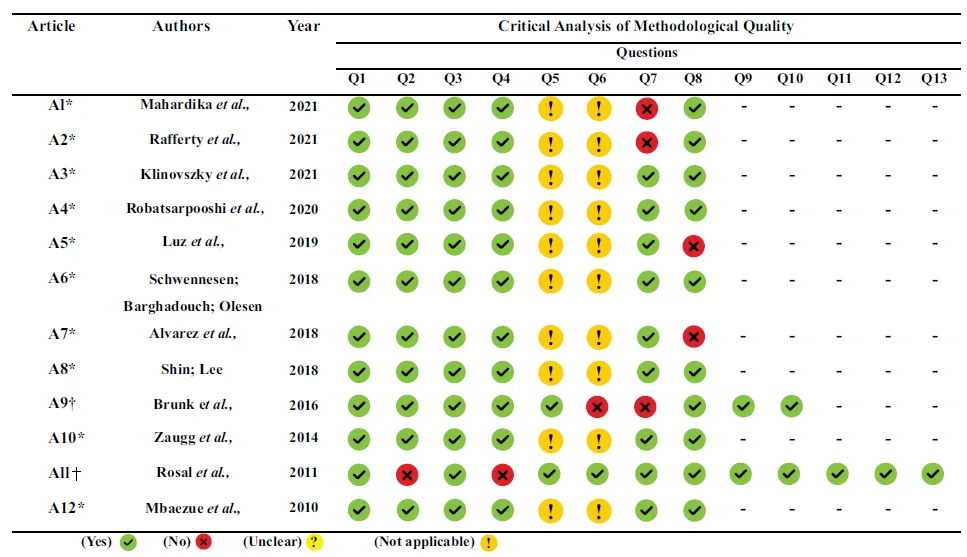
Critical analysis of methodological quality of the studies according to the JBI. Note: *Cross-sectional studies checklist † qualitative research checklist 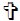 randomized controlled trials checklist.

**Table 1:** Characterization of articles included in the systematic review sample.

**Authors and Year**	**Country/ Language**	**Objective**	**Sample**	**Setting**	**Instrument Used**	**Main Results**	Level of Evidence*
Mahardika *et al.*, 2021 [21]	Indonesia English	Analyze the key determinant for type 2 diabetes patients performing blood glucose self-monitoring during COVID-19.	169 individuals with T2DM and age >15 years.	A public health center	Questionnaires	A statistically significant correlation was found between education level (*p*=0.000), HL (*p*=0.001), and SMBG among type 2 diabetes patients during COVID-19. High HL individuals are 3.051 times more likely [1.326-7.019] to perform SMBG [1.137-5.436], while those with high anxiety have a 0.171 times lower chance.	2C
Rafferty *et al.*, 2021 [22]	USA English	Examine self-care and clinical care in adults with diabetes across three health literacy dimensions.	4,397 people with DM.	Centers for Disease Control and Prevention	Instrument developed by the author with 3 triggering questions	Most patients found the three HL tasks easy. However, 5.9% struggled to obtain health advice, 10.7% had difficulty understanding healthcare information, and 12.0% had trouble with written health information. Those who struggled to obtain health advice were over 30% less likely to perform SMBG.	2C
Klinovszky, Zipernovszky, Buzás, 2021 [23]	Hungary English	Explore Health Functional Literacy (HFL) and numeracy skills in insulin-treated type 2 diabetes patients and their impact on self-care activities.	102 patients with T2DM on insulin therapy.	Three diabetes associations	B-TOFHLA and DNT-15	The analysis showed a weak positive correlation between B-TOFHLA scores and daily SMBG frequency (rs (99) = 0.370, *p* < 0.001) and a moderate positive correlation between DNT 15 scores and daily SMBG frequency (rs (100) = 0.473, *p* < 0.001).	2C
RobatSarpooshi *et al.*, 2020 [24]	Iran English	Investigate the relationship between Health Functional Literacy and self-care behaviors in diabetic patients to reduce complications, improve quality of life, and lessen the economic burden on families and society.	400 individuals with T2DM, aged ≥ 30 years, and a minimum diagnosis of 6 months.	Public and private health centers and diabetes clinics	HELIA	There were significant relationships between various dimensions of HL (reading, access, understanding, appraisal, use, and decision-making) and domains of self-care behavior (healthy eating, physical activity, blood glucose monitoring, and appropriate medication consumption) with *p* <0.05.	2C
Luz *et al.*, 2019 [25]	Brazil English	Associate the level of Health Functional Literacy and self-care with Diabetes.	97 individuals with DM, aged over 30 years, and with ≥ 6 months of follow-up.	Reference center for diabetes care	B-TOFHLA and QAD	There was an association between inadequate HL and glucose monitoring at the recommended frequency, with a statistically significant difference (*p* < 0.001).	2C
Schwennesen, Barghadouch, Olesen, 2018 [26]	Denmark English	Investigate health functional literacy and self-care among visually impaired type 1 diabetes patients in Denmark.	1,425 individuals with type 1 diabetes (T1DM), including 38 visually impaired.	Specialized diabetes clinic	HLQ and SDSCA	Visually impaired patients, older and with longer diabetes duration, were more likely to live alone. They showed lower health literacy in 'Finding good health information' and 'Understanding health information,' which monitored blood sugar less frequently than sighted individuals, but exhibited better self-care in foot examinations.	2C
Alvarez *et al.*, 2018 [27]	USA English	Measure the association between health functional literacy and clinical outcomes and patient-reported outcomes in non-insulin-treated DM2 patients.	448 individuals with T2DM, aged ≥ 30 years, and A1C level between 6.5 and 9.5% in the previous 6 months.	University health unit	NVS	Patients with limited HL had poorer glycemic control compared to those with adequate HL (A1C 7.7 ± 1.1% *vs.* 7.5 ± 1.0%, *p* = 0.016), despite more frequent use of SMBG testing (daily SMBG testing 49.3% *vs.* 30.7%, *p* = 0.001). The difference in doctor advice frequency for SMBG testing between limited and adequate HL groups was not significant (*p* = 0.68).	2C
Shin; Lee, 2018 [28]	South Korea English	Examine the relationships between health functional literacy and diabetes self-care behaviors (diet, physical exercise, foot care, and blood glucose monitoring) through empowered diabetes education.	136 individuals with T2DM aged ≥60 years	Three community health centers	HLS, DES-28, and SDSCA	Diabetes education recipients showed significantly higher scores for empowerment and self-care behaviors (diet, exercise, foot care, and SMBG) than non-recipients. The indirect effects of HL on self-care behaviors through empowerment were significant for diet and physical exercise but not for foot care and blood glucose monitoring.	2C
Brunk *et al.*, 2016 [29]	USA English	Evaluate the feasibility of adapting a patient-centered educational intervention for type 2 diabetes self-management to a Hispanic population with low health literacy skills.	9 people with T2DM	Rural community health center	Focus group with record of patient feedback	All patients expressed a lack of knowledge about the basic principles of glycemic control. Some patients were not using SMBG and thus gained new awareness of their glycemic status and the importance of monitoring their blood glucose levels.	2C
Zaugg *et al.*, 2014 [30]	USA English	Assess diabetes numeracy using the DNT-15 in rural diabetes patients.	194 individuals with T1DM and T2DM, aged 18 or older, and diagnosed for more than 12 months	Local primary care practices (PCP) and diabetes center	DNT-15	In the diabetes center, 63% (79 out of 125) adjusted medications based on glucose readings, compared to 28% (19 out of 69) in primary care (*p* <0.001). About 44% (55 out of 125) of diabetes center patients calculated insulin doses based on carbohydrates versus 7% (5 out of 69) of primary care patients (*p* <0.001). A significant majority (79.8%) of diabetes center patients completed diabetes education, compared to 37.7% in primary care. Among T2DM patients, 54% received diabetes education, contrasting with 88.5% of T1DM patients. Patients who underwent diabetes education had significantly higher numeracy scores than those who did not (*p* <0.001).	2C
Rosal *et al.*, 2011 [31]	USA English	Test a theory-based, literacy, and culturally adapted self-management intervention, Latinos en Control, to improve glycemic control among low-income Latinos with type 2 diabetes.	Two hundred fifty-two individuals with T2DM, aged 18 or older, documented diagnosis, last HbA1c within the previous 7 months, divided into 2 groups of 124 and 128.	Five community health centers	Behavioral measures included telephone-based 24-hour recalls. Diabetes knowledge was assessed using the Audit of Diabetes Knowledge. Additionally, a 17-item tool developed by the research team evaluated self-efficacy.	At 4 months, there was a significant difference in HbA1c reduction between groups (intervention -0.88 *vs.* control -0.35, *p* < 0.01), which decreased and lost significance by 12 months (intervention -0.46 *vs.* control -0.20, *p* = 0.293). The intervention significantly improved diabetes knowledge at 12 months (*p* = 0.001) and increased SMBG (*p* = 0.02). Moreover, the proportion of patients performing blood glucose measurements two or more times daily significantly rose in the intervention group (from 59% to 84.2% and 81.5% at 4 and 12 months, respectively), compared to the control group (from 55.7% to 62.1-63.6% at 4 and 12 months, respectively) (*p* = 0.02 and *p* = 0.023 for changes at 4 and 12 months).	1B
Mbaezue *et al.*, 2010 [32]	USA English	Examine the relationship between health literacy and blood glucose self-monitoring.	189 individuals with DM, aged between 18 and 65 years.	Hospital clinic	B-TOFHLA	The majority (60.9%) of patients were assessed with adequate HL, and 90.9% reported testing blood sugar at least once daily. Although HL was associated with recording blood sugar tests (*p* = 0.049), no statistically significant relationship was found between HL and the frequency of SMBG. Individuals who reported having DM for more than 10 years were less likely to perform SMBG (odds ratio, 0.33; 95% CI, 0.11-0.99).	2C

**Note:** *(Oxford, 2009) [16].

## LIST OF ABBREVIATIONS

DM: Diabetes Mellitus;
IDF: International Diabetes Federation
T2DM: Type 2 Diabetes Mellitus
SMBG: Self-monitoring of Blood Glucose
CGM: Continuous Glucose Monitoring
HL: Health Literacy

## STANDARDS OF REPORTING

PRISMA guidelines were followed.

## SUPPLEMENTARY MATERIAL

PRISMA checklist is available on the publisher’s website along with the published article.

## References

[1] Ogurtsova K., Guariguata L., Barengo N.C., Ruiz P.L.D., Sacre J.W., Karuranga S., Sun H., Boyko E.J., Magliano D.J. (2022). IDF diabetes atlas: Global estimates of undiagnosed diabetes in adults for 2021.. Diabetes Res. Clin. Pract..

[2] American Diabetes Association (2021). Introduction: Standards of medical care in diabetes—2021.. Diabetes Care.

[3] American Diabetes Association (2021). Glycemic targets: Standards of Medical care in diabetes—2021.. Diabetes Care.

[4] Maiorino M.I., Signoriello S., Maio A., Chiodini P., Bellastella G., Scappaticcio L., Longo M., Giugliano D., Esposito K. (2020). Effects of continuous glucose monitoring on metrics of glycemic control in diabetes: A systematic review with meta-analysis of randomized controlled trials.. Diabetes Care.

[5] Ong W.M., Chua S.S., Ng C.J. (2014). Barriers and facilitators to self-monitoring of blood glucose in people with type 2 diabetes using insulin: A qualitative study.. Patient Prefer. Adherence.

[6] Fisher W.A., Kohut T., Schachner H., Stenger P. (2011). Understanding self-monitoring of blood glucose among individuals with type 1 and type 2 diabetes: An information-motivation-behavioral skills analysis.. Diabetes Educ..

[7] Broos B., Charleer S., Bolsens N., Moyson C., Mathieu C., Gillard P., De Block C. (2021). Diabetes knowledge and metabolic control in type 1 diabetes starting with continuous glucose monitoring: Future-peak.. J. Clin. Endocrinol. Metab..

[8] Sørensen K., Van den Broucke S., Fullam J., Doyle G., Pelikan J., Slonska Z., Brand H. (2012). Health literacy and public health: A systematic review and integration of definitions and models.. BMC Public Health.

[9] (1999). Health literacy: Report of the council on scientific affairs. Ad Hoc committee on health literacy for the council on scientific affairs, american medical association.. JAMA.

[10] Rothman R.L., Housam R., Weiss H., Davis D., Gregory R., Gebretsadik T., Shintani A., Elasy T.A. (2006). Patient understanding of food labels: The role of literacy and numeracy.. Am. J. Prev. Med..

[11] Osborn C.Y., Cavanaugh K., Kripalani S. (2010). Strategies to address low health literacy and numeracy in diabetes.. Clin. Diabetes.

[12] Shiyanbola O.O., Unni E., Huang Y.M., Lanier C. (2018). The association of health literacy with illness perceptions, medication beliefs, and medication adherence among individuals with type 2 diabetes.. Res. Social Adm. Pharm..

[13] Bressan V., Bagnasco A., Aleo G., Timmins F., Barisone M., Bianchi M., Pellegrini R., Sasso L. (2017). Mixed-methods research in nursing – A critical review.. J. Clin. Nurs..

[14] Alonso PC, Schünemann HJ, Moberg J, Brignardello RP, Akl EA, Davoli M (2016). Grade evidence to decision (EtD) frameworks: A systematic and transparent approach to making well informed healthcare choices.. BMJ.

[15] Zhang Y., Coello A.P., Guyatt G.H., Nuñez Y.J.J., Akl E.A., Hazlewood G., Hernandez P.H., Ikobaltzeta E.I., Qaseem A., Williams J.W., Tugwell P., Flottorp S., Chang Y., Zhang Y., Mustafa R.A., Rojas M.X., Schünemann H.J. (2019). Grade guidelines: 19. Assessing the certainty of evidence in the importance of outcomes or values and preferences—risk of bias and indirectness.. J. Clin. Epidemiol..

[16] Aromataris E, Lockwood C, Porritt K, Pilla B, Jordan Z (2024). JBI manual for evidence synthesis.. https://jbi-global-wiki.refined.site/space/MANUAL.

[17] Page M.J., McKenzie J.E., Bossuyt P.M., Boutron I., Hoffmann T.C., Mulrow C.D., Shamseer L., Tetzlaff J.M., Akl E.A., Brennan S.E., Chou R., Glanville J., Grimshaw J.M., Hróbjartsson A., Lalu M.M., Li T., Loder E.W., Wilson M.E., McDonald S., McGuinness L.A., Stewart L.A., Thomas J., Tricco A.C., Welch V.A., Whiting P., Moher D. (2021). The PRISMA 2020 statement: An updated guideline for reporting systematic reviews.. BMJ.

[18] Lizarondo L., Stern C., Carrier J., Godfrey C., Rieger K., Salmond S. (2024). Mixed methods systematic reviews.. https://synthesismanual.jbi.global.

[19] (2009). Oxford centre for evidence-based medicine.. http://www.cebm.net/oxford-centre-evidence-based-medicine-levels-evidence-march-2009.

[20] Munn Z., Barker T.H., Moola S., Tufanaru C., Stern C., McArthur A. (2020). Methodological quality of case series studies: An introduction to the JBI critical appraisal tool.. JBI Evid Synth..

[21] Mahardika I.M.R., Suyasa G.P.D., Kamaryati N.P., Wulandari S.K. (2021). Health literacy is strongest determinant on self-monitoring blood glucose (SMBG) type 2 DM patients during COVID-19 pandemic at public health centre in tabanan regency.. Int. J. Healthc. Med. Sci..

[22] Rafferty A.P., Winterbauer N.L., Luo H., Bell R.A., Little N.R.G. (2021). Diabetes self-care and clinical care among adults with low health literacy.. J. Public Health Manag. Pract..

[23] Klinovszky A., Zipernovszky P.O., Buzás N. (2021). Building a house of skills—A study of functional health literacy and numeracy among patients with type 2 diabetes in hungary.. Int. J. Environ. Res. Public Health.

[24] RobatSarpooshi D., Mahdizadeh M., Siuki A.H., Haddadi M., Robatsarpooshi H., Peyman N., Mahdizadeh M, Siuki A.H, Haddadi M, Robatsarpooshi H, Peyman N. (2020). The relationship between health literacy level and self-care behaviors in patients with diabetes.. Patient Relat. Outcome Meas..

[25] Luz G.O.A., Alves D.D.D.A., Costa H.K.D.S., Filho S.J.C., Stratmann P.F., Souza M.A.D.O., Santos I.C.R.V. (2019). Association between functional health literacy and self-care with diabetes mellitus.. Think Nursing.

[26] Schwennesen N., Barghadouch A., Olesen K. (2019). Health literacy and self-care among visually impaired people with type 1 diabetes in denmark.. Chronic Illn..

[27] Alvarez PM, Young LA, Mitchell M, Blakeney TG, Buse JB, Vu MB (2018). Health literacy, glycemic control, and physician-advised glucose self-monitoring use in type 2 diabetes.. Diabetes Spectr.

[28] Shin K.S., Lee E.H. (2018). Relationships of health literacy to self-care behaviors in people with diabetes aged 60 and above: Empowerment as a mediator.. J. Adv. Nurs..

[29] Brunk D.R., Taylor A.G., Clark M.L., Williams I.C., Cox D.J. (2017). A culturally appropriate self-management program for Hispanic adults with type 2 diabetes and low health literacy skills.. J. Transcult. Nurs..

[30] Zaugg S.D., Dogbey G., Collins K., Reynolds S., Batista C., Brannan G., Shubrook J.H. (2014). Diabetes numeracy and blood glucose control: Association with type of diabetes and source of care.. Clin. Diabetes.

[31] Rosal M.C., Ockene I.S., Restrepo A., White M.J., Borg A., Olendzki B., Scavron J., Candib L., Welch G., Reed G. (2011). Randomized trial of a literacy-sensitive, culturally tailored diabetes self-management intervention for low-income latinos: Latinos en control.. Diabetes Care.

[32] Mbaezue N., Mayberry R., Gazmararian J., Quarshie A., Ivonye C., Heisler M. (2010). The impact of health literacy on self-monitoring of blood glucose in patients with diabetes receiving care in an inner-city hospital.. J. Natl. Med. Assoc..

[33] Jiao Y., Lin R., Hua X., Churilov L., Gaca M.J., James S., Clarke P.M., O’Neal D., Ekinci E.I. (2022). A systematic review: Cost-effectiveness of continuous glucose monitoring compared to self-monitoring of blood glucose in type 1 diabetes.. Endocrinol. Diabetes Metab..

[34] Cutruzzolà A., Irace C., Parise M., Fiorentino R., Tripodi P.P.F., Ungaro S., Babinsky V., Gnasso A. (2020). Time spent in target range assessed by self-monitoring blood glucose associates with glycated hemoglobin in insulin treated patients with diabetes.. Nutr. Metab. Cardiovasc. Dis..

[35] Evans EI, Pincus KJ, Seung H, Rochester CDE (2024). Health literacy of patients using continuous glucose monitoring.. J Am Pharm Assoc.

[36] Cavanaugh K., Wallston K.A., Gebretsadik T., Shintani A., Huizinga M.M., Davis D., Gregory R.P., Malone R., Pignone M., DeWalt D., Elasy T.A., Rothman R.L. (2009). Addressing literacy and numeracy to improve diabetes care: Two randomized controlled trials.. Diabetes Care.

[37] Osborn C.Y., Bains S.S., Egede L.E. (2010). Health literacy, diabetes self-care, and glycemic control in adults with type 2 diabetes.. Diabetes Technol. Ther..

[38] Osborn C.Y., Cavanaugh K., Wallston K.A., Rothman R.L. (2010). Self-efficacy links health literacy and numeracy to glycemic control.. J. Health Commun..

[39] Koh Y.S., AshaRani P.V., Devi F., Roystonn K., Wang P., Abdin E., Sum C.F., Lee E.S., Chong S.A., Subramaniam M. (2024). Diabetes self-care behaviors in Singapore and their associations with patients’ characteristics and health literacy.. Sci. Diabetes Self Manag. Care.

[40] Kirk J.K., Stegner J. (2010). Self-monitoring of blood glucose: Practical aspects.. J. Diabetes Sci. Technol..

[41] Pimentel J de O (2021). Self-care of users with diabetes mellitus entered into a program of capillary blood glucose self-monitoring.. Rev Pesqui Cuid é Fundam.

[42] Moura N.S., Lopes B.B., Teixeira J.J.D., Oriá M.O.B., Vieira N.F.C., Guedes M.V.C. (2019). Literacy in health and self-care in people with type 2 diabetes mellitus.. Rev. Bras. Enferm..

[43] Turrin K.B., Trujillo J.M. (2019). Effects of diabetes numeracy on glycemic control and diabetes self-management behaviors in patients on insulin pump therapy.. Diabetes Ther..

